# Health-related quality of life, work productivity, and indirect costs among patients with irritable bowel syndrome with diarrhea

**DOI:** 10.1186/s12955-017-0611-2

**Published:** 2017-02-14

**Authors:** Jessica L. Buono, Robyn T. Carson, Natalia M. Flores

**Affiliations:** 1 Allergan plc, Jersey City, NJ USA; 2 Kantar Health, Foster City, CA USA

**Keywords:** Irritable bowel syndrome with diarrhea, IBS-D, Health-related quality of life, HRQoL, Work productivity impairment, Activity impairment, Indirect costs, National Health and Wellness Survey

## Abstract

**Background:**

Irritable bowel syndrome (IBS) affects 10–15% of adults in the US, and is associated with significant impairment in health-related quality of life (HRQoL); however, information specific to the diarrhea subtype (IBS-D) is lacking. We assessed the impact of IBS-D on HRQoL, work productivity, and daily activities, and the associated indirect costs, among a sample of the US population.

**Methods:**

Respondents (≥18 years) from the 2012 US National Health and Wellness Survey who reported an IBS-D diagnosis by a physician or symptoms consistent with Rome II criteria for IBS-D were identified as having IBS-D. Controls included respondents without IBS-D or inflammatory bowel disease. HRQoL was assessed via the Short Form 36 Health Survey version 2 questionnaire and summarized into Mental and Physical Component Summary (MCS; PCS) scores and a Short Form-6 dimension (SF-6D) utility score. Work and activity impairment were assessed via the Work Productivity and Activity Impairment Questionnaire: General Health version (WPAI:GH), which measures absenteeism, presenteeism, overall work productivity loss, and daily activity impairment. Indirect costs were calculated using unit cost data from the Bureau of Labor Statistics and variables from the WPAI:GH. Generalized linear models were used to examine differences in health outcomes between respondents with IBS-D and controls, controlling for demographic and health characteristics.

**Results:**

In total, 66,491 respondents (1102 IBS-D; 65,389 controls) were analyzed. Mean age was 48.7 years; 50% were female. Compared with controls, the IBS-D cohort reported significantly lower HRQoL (mean MCS: 45.16 vs. 49.48; *p* < 0.001; mean PCS: 47.29 vs. 50.67; *p* < 0.001; mean SF-6D: 0.677 vs. 0.741; *p* < 0.001) and greater absenteeism (5.1% vs. 2.9%; *p* = 0.004), presenteeism (17.9% vs. 11.3%; *p* < 0.001), overall work productivity loss (20.7% vs. 13.2%; *p* < 0.001), and activity impairment (29.6% vs. 18.9%; *p* < 0.001). Respondents with IBS-D also incurred an estimated $2486 more in indirect costs ($7008 vs. $4522; *p* < 0.001).

**Conclusions:**

Compared with controls, IBS-D is associated with significantly lower HRQoL, greater impairments in work and daily activities, and higher indirect costs, imposing a substantial burden on patients and employers. These findings suggest a significant unmet need exists for effective IBS-D treatments.

## Background

Irritable bowel syndrome (IBS) is a chronic functional gastrointestinal disorder characterized by abdominal pain or discomfort associated with altered bowel habits, in the absence of discernable organic disease such as microscopic colitis and inflammatory bowel disease (IBD) [1, 2]. IBS affects 10–15% of adults in the US, and is a common diagnosis in both gastroenterology and primary care practices [3]. IBS is classified into subtypes based on predominant stool pattern, including IBS with diarrhea (IBS-D), IBS with constipation (IBS-C), and IBS with mixed bowel patterns of constipation and diarrhea [2]. IBS-D is estimated to account for approximately one-third of IBS cases [4].

As the symptom profile and management strategies for each IBS subtype differ, it is important to understand the burden of illness associated with each subtype. While prior studies have assessed the impact of IBS-C on health-related quality of life (HRQoL) and work and activity impairment [5, 6], information specific to the burden of IBS-D is limited [7].

Among IBS overall, the symptom burden experienced by patients is associated with significant impairments in HRQoL [3, 7, 8]. In a systematic review, patients with IBS had significantly lower scores compared with controls across every domain of the Short Form 36 Health Survey (SF-36), a measure for assessing HRQoL – including both physical and emotional role functioning – as well as mental health [7]. In a further study, impairments in HRQoL experienced by IBS patients were comparable to or greater than HRQoL impairments seen among patients with other chronic diseases, with IBS patients reporting lower HRQoL based on several domains of the SF-36, including bodily pain, social functioning, and mental health, in comparison with patients with asthma, gastroesophageal reflux disease (GERD), and migraine [9]. For IBS-D specifically, the burden of symptoms, including abdominal pain, loose and watery stools, cramping, and bloating, can significantly impact patients physically, emotionally, and socially. In a recent survey of 1000 IBS-D patients in the US, over half of IBS-D patients reported their symptoms were extremely or very bothersome [10].

While information specific to IBS-D is limited, IBS in general is also associated with significant impairments in work productivity. In a 2015 survey conducted by the American Gastroenterological Association, IBS patients reported that their symptoms interfered with work productivity an average of 9 days per month and that they missed an average of 2 days of work per month [10]. Indirect costs (e.g. loss of work and reduced productivity) of IBS have been estimated at up to $20 billion annually in the US, with an estimated annual cost per patient of $9933 (in 2012 US dollars) [3, 7].

Given that data specific to the burden associated with IBS-D are limited, the objective of this study was to quantify the burden of disease among individuals with IBS-D relative to those without IBS-D, with regard to HRQoL, work productivity loss and daily activity impairment, and associated indirect costs among a sample of the US population.

## Methods

### Data source

This study utilized data from the 2012 US National Health and Wellness Survey (NHWS), a self-administered, internet-based general health questionnaire, from a sample of adults in the US (aged ≥18 years). Adults were identified for inclusion in the survey through a web-based opt-in consumer panel of pre-recruited individuals who consented to participate in research. Stratified random sampling (with stratification by gender and age) was used to ensure the demographic composition of the survey population was representative of the US adult population based on data from the US Census.

### Sample population

**Fig. 1:**
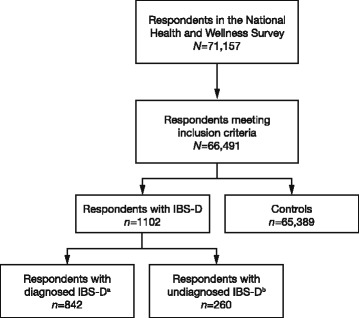
Respondent identification. ^a^Defined as respondents who self-reported a diagnosis of IBS-D by a physician and reported diarrhea as the predominant bowel symptom. ^b^Defined as respondents who reported symptoms consistent with the Rome II criteria for an IBS-D diagnosis but did not self-report a diagnosis of IBS-D by a physician. *Abbreviations*: *IBS-D* irritable bowel syndrome with diarrhea

The initial study sample included all respondents who completed the 2012 NHWS (Fig. 1). Respondents with IBS-D were classified into two categories: 1) “diagnosed” IBS-D, if they self-reported a diagnosis of IBS by a physician and reported diarrhea as the predominant bowel symptom; or 2) “undiagnosed IBS-D”, if they reported symptoms consistent with the Rome II criteria for IBS-D but did not self-report a diagnosis of IBS-D by a physician. Controls included respondents who did not self-report experiencing IBS symptoms (any subtype) or subsequent diagnoses, did not meet symptom criteria for IBS according to the Rome II criteria, and did not self-report experiencing IBD or subsequent diagnoses.

### Study design

This study was an analysis of cross-sectional survey data assessing HRQoL and work productivity and daily activity impairment among respondents with IBS-D relative to controls. HRQoL was evaluated based on the Medical Outcomes SF-36 version 2 (SF-36v2) [11], which includes a Physical Component Summary (PCS) score and a Mental Component Summary (MCS) score (range: 0–100). Higher scores indicate better quality of life. Health utility scores were derived from the SF-36v2 by calculating the Short Form-6 dimension (SF-6D) score, a health state classification measure ranging from 0.0 (death) to 1.0 (best health state).

Work productivity and activity impairment were assessed based on the Work Productivity and Activity Impairment Questionnaire: General Health version (WPAI:GH) [12]. The WPAI:GH consists of six items measuring four domains: 1) absenteeism (percentage of work time missed due to health problems in the past 7 days, calculated as: [hours missed due to health problems/(hours missed due to health problems + hours worked)] x 100); 2) presenteeism (percentage of impairment experienced at work due to health problems in the past 7 days, calculated as: [degree health affected productivity while working/10] x 100); 3) overall work productivity loss (absenteeism plus presenteeism, calculated as: [absenteeism + (hours worked x presenteeism)] x 100); and 4) daily activity impairment (percentage of impairment in daily activities due to health problems in the past 7 days, calculated as: [degree health affected daily activities/10] x 100). Only respondents who reported being employed (full or part time) provided data for absenteeism, presenteeism, and overall work productivity loss; all respondents provided data for daily activity impairment. All domain scores are expressed as percentages, with higher percentages indicating greater work productivity loss and activity impairment. Days of work missed annually (for employed participants only) were calculated using reported work hours missed based on the WPAI:GH, assuming a 40-hour work week and 50 weeks worked annually.

Estimated annual indirect costs were calculated by integrating data from the Bureau of Labor Statistics and the WPAI:GH, with 2012 median weekly wages by age and sex obtained from the Bureau of Labor Statistics applied as unit costs to work productivity variables (absenteeism and presenteeism) from the WPAI:GH. The number of hours missed due to absenteeism and presenteeism were calculated for each respondent and multiplied by their associated estimated hourly wage, based on age and gender. These figures were annualized by multiplying by 50 work weeks in a year, and combined to estimate the total indirect costs. Costs are presented in 2012 US dollars.

### Statistical analysis

Differences in demographic and health characteristics between diagnosed respondents with IBS-D, undiagnosed respondents with IBS-D, and controls were examined utilizing one-way analysis of variance (for continuous variables) and Chi-square tests (for categorical variables). When comparing more than two groups, appropriate pairwise comparisons were used. For analysis of differences in HRQoL, work productivity and activity impairment, and indirect costs, diagnosed and undiagnosed respondents with IBS-D were combined into a single category of “IBS-D”.

Generalized linear models were used to assess differences in HRQoL, work productivity, and daily activity impairment between respondents with IBS-D (undiagnosed and diagnosed combined) and controls, controlling for all demographic and health characteristics identified *a priori*, including age, gender, ethnicity, income, education, body mass index category, smoking status, alcohol use, exercise activity, and Charlson Comorbidity Index (CCI). Estimated means and standard errors are reported. For all measures, differences were considered significant at *p* < 0.05.

## Results

### Demographics and health characteristics

The sample population included a total of 66,491 respondents: 1102 with IBS-D (842 diagnosed, 260 undiagnosed) and 65,389 controls (Fig. 1). In the overall sample, mean age was 48.7 years, 50% were female, and 54% were employed (Table 1). Respondents with IBS-D were more likely to be female (diagnosed: 60.6%; undiagnosed: 65.0%; controls: 50.0%; *p* < 0.001) and had a higher CCI score than controls (diagnosed: 0.72; undiagnosed: 0.75; controls: 0.41; *p* < 0.001).

**Table 1:** Demographic and health characteristics of respondents with IBS-D (diagnosed and undiagnosed) and controls

	Total *n* = 66,491	IBS-D, diagnosed *n* = 842	IBS-D, undiagnosed *n* = 260	Controls *n* = 65,389	*p-value*
Age, mean (SD)	48.72 (16.61)	50.39 (15.79)_a_	43.05 (14.40)_b_	48.72 (16.62)_c_	<0.001
Charlson Comorbidity Index, mean (SD)	0.42 (0.99)	0.72 (1.23)_a_	0.75 (1.50)_a_	0.41 (0.98)_b_	<0.001
Female, *n* (%)	33377 (50.2)	510 (60.6)_a_	169 (65.0)_a_	32698 (50.0)_b_	
Race/ethnicity, *n* (%)					<0.001
Non-Hispanic White	47700 (71.7)	712 (84.6)_a_	180 (69.2)_b_	46808 (71.6)_b_	
Non-Hispanic Black	7857 (11.8)	31 (3.7)_a_	28 (10.8)_b_	7798 (11.9)_b_	
Hispanic	5603 (8.4)	49 (5.8)_a_	27 (10.4)_b_	5527 (8.5)_b_	
Other ethnicity	5331 (8.0)	50 (5.9)_a_	25 (9.6)_b_	5256 (8.0)_b_	
Education, *n* (%)					0.069
< 4-year degree	39657 (59.6)	496 (58.9)_a_	173 (66.5)_b_	38988 (59.6)_a_	
≥ 4-year degree	26834 (40.4)	346 (41.1)_a_	87 (33.5)_b_	26401 (40.4)_a_	
Annual household income, *n* (%)					0.008
< $25 k	12750 (19.2)	161 (19.1)_a_	65 (25.0)_b_	12524 (19.2)_a_	
$25–50 k	17775 (26.7)	215 (25.5)_a_	80 (30.8)_a_	17480 (26.7)_a_	
$50–75 k	13037 (19.6)	189 (22.4)_a_	54 (20.8)_a,b_	12794 (19.6)_b_	
≥ $75 k	17369 (26.1)	214 (25.4)_a_	46 (17.7)_b_	17109 (26.2)_a_	
Declined to answer	5560 (8.4)	63 (7.5)_a_	15 (5.8)_a_	5482 (8.4)_a_	
Labor force participation, *n* (%)					0.424
Yes	35636 (53.6)	433 (51.4)_a_	142 (54.6)_a_	35061 (53.6)_a_	
No	30855 (46.4)	409 (48.6)_a_	118 (45.4)_a_	30328 (46.4)_a_	
Body mass index, *n* (%)					<0.001
Underweight (<18.5)	1231 (1.9)	11 (1.3)_a_	3 (1.2)_a_	1217 (1.9)_a_	
Normal weight (18.5–24.99)	20894 (31.4)	228 (27.1)_a_	76 (29.2)_a,b_	20590 (31.5)_b_	
Overweight (25–29.99)	21347 (32.1)	243 (28.9)_a_	68 (26.2)_a_	21036 (32.2)_b_	
Obese (≥30)	21265 (32.0)	341 (40.5)_a_	111 (42.7)_a_	20813 (31.8)_b_	
Unknown	1754 (2.6)	19 (2.3)_a_	2 (0.8)_a_	1733 (2.7)_a_	

For pairwise comparisons, values in the same row not sharing the same subscript letter (a, b, or c) are significantly different at *p* < 0.05

*Abbreviations*: *IBS-D* irritable bowel syndrome with diarrhea, *SD* standard deviation

Among respondents who reported a diagnosis of IBS-D, the majority were diagnosed by a primary care physician (51.5%) or a gastroenterologist (40.0%), with the remainder reporting being diagnosed by a nurse practitioner/physician assistant (2.9%), obstetrician/gynecologist (1.5%), or other practitioner (4.1%). The average time since diagnosis of IBS-D was 11 years. Of diagnosed IBS-D respondents, 28.8% reported current use of prescription medication, and 33.8% reported the use of an over-the-counter (OTC) medication. Dicyclomine hydrochloride was the most commonly reported prescription medicine (16.7%), while loperamide was the most commonly reported OTC medication (43.6%). Among the diagnosed respondents, 48.0% reported having symptoms at least 2 to 3 times per week, and 55.8% considered their symptoms to be bothersome to extremely bothersome (Table 2).

**Table 2:** Frequency, bothersomeness, and severity of symptoms among respondents with IBS-D (diagnosed and undiagnosed)

	Response	Respondents
		*n* = 875^a^
IBS severity, *n* (%)	Mild	325 (37.1)
	Moderate	441 (50.4)
	Severe	109 (12.5)
Frequency of IBS symptoms, *n* (%)	Daily	120 (13.7)
	4–6 times a week	114 (13.0)
	2–3 times a week	186 (21.3)
	Once a week	112 (12.8)
	2–3 times a month	204 (23.3)
	Once a month or less often	139 (15.9)
Bothersomeness of IBS symptoms, *n* (%)	Not at all bothersome	51 (5.8)
	Somewhat bothersome	335 (38.3)
	Bothersome	241 (27.5)
	Very bothersome	162 (18.5)
	Extremely bothersome	86 (9.8)

^a^Not all IBS-D respondents provided a response

*Abbreviations*: *IBS* irritable bowel syndrome, *IBS-D* irritable bowel syndrome with diarrhea

### Health-related quality of life

As minimal differences in demographics (and outcomes, including HRQoL and work productivity and activity impairment [data not shown]) were observed between the diagnosed and undiagnosed IBS-D cohorts, these two groups were combined into a single category of “IBS-D” for all subsequent analyses. Respondents with IBS-D reported significantly lower HRQoL versus controls. Mean MCS (45.16 vs. 49.48; *p* < 0.001) and PCS (47.29 vs. 50.67; *p* < 0.001) scores were significantly lower for individuals with IBS-D than for controls, after controlling for demographic and health characteristics (Table 3). Mean SF-6D scores for respondents with IBS-D were also significantly lower than in the control group, with a mean difference of 0.064 (0.677 vs. 0741; *p* < 0.001) after adjusting for covariates (Table 3). For the eight subscales that make up the SF-36v2, respondents with IBS-D had significantly lower scores than controls in all domains (*p* < 0.001 vs. controls for all domains) [Table 3].

**Table 3:** SF-36v2 and SF-6D scores among respondents with IBS-D (diagnosed and undiagnosed) and controls

Score, mean (SE)	IBS-D	Controls	*p-value* ^a^
	*n* = 1102	*n* = 65,389	
*SF-36v2 summary scores*			
MCS	45.16 (0.29)	49.48 (0.04)	<0.001
PCS	47.29 (0.25)	50.67 (0.03)	<0.001
*SF-36v2 subscales*			
Bodily pain	45.66 (0.28)	50.20 (0.04)	<0.001
General health	45.88 (0.27)	50.89 (0.04)	<0.001
Mental health	45.32 (0.30)	49.40 (0.04)	<0.001
Physical functioning	47.76 (0.27)	50.09 (0.04)	<0.001
Role limitations due to emotional problems	46.11 (0.30)	49.47 (0.04)	<0.001
Role limitations due to physical health	47.01 (0.27)	50.25 (0.03)	<0.001
Social functioning	45.35 (0.28)	49.75 (0.04)	<0.001
Vitality	45.74 (0.28)	50.65 (0.04)	<0.001
*SF-6D score*	0.667 (0.004)	0.741 (0.000)	<0.001

^a^Analyses adjusted for age, gender, ethnicity, income, education, body mass index, smoking status, alcohol use, exercise activity, and Charlson Comorbidity Index

*Abbreviations*: *IBS-D* irritable bowel syndrome with diarrhea, *MCS* Mental Component Summary, *PCS* Physical Component Summary, *SE* standard error, *SF-36v2* Short Form 36 Health Survey version 2, *SF-6D* Short Form 6-dimension

### Work productivity and activity impairment

**Fig. 2:**
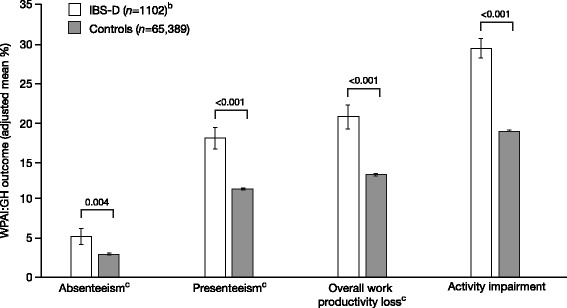
Work productivity and activity impairment among respondents with IBS-D (diagnosed and undiagnosed) and controls^a^. ^a^Analyses adjusted for age, gender, ethnicity, income, education, body mass index, smoking status, alcohol use, exercise activity, and Charlson Comorbidity Index. ^b^Includes diagnosed and undiagnosed respondents. ^c^Includes employed respondents only (*n* = 557 for respondents with IBS-D, *n* = 33,414 for controls). *Abbreviations*: *IBS-D* irritable bowel syndrome with diarrhea, *WPAI:GH* Work Productivity and Activity Impairment: General Health version

Compared with controls, respondents with IBS-D missed significantly more work (absenteeism 5.1% vs. 2.9%; *p* = 0.004), experienced higher levels of presenteeism while at work (17.9% vs. 11.3%; *p* < 0.001), and had greater overall work productivity loss (20.7% vs. 13.2%; *p* < 0.001) and higher daily activity impairment (29.6% vs. 18.9%; *p* < 0.001) [Fig. 2]. Individuals with IBS-D also missed significantly more work days annually compared with controls, with a mean difference of 3.9 days lost per year (10.1 vs. 6.2; *p* = 0.031).

### Indirect costs

**Fig. 3:**
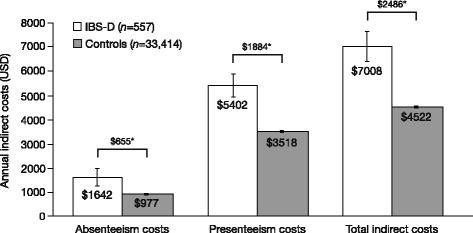
Annual indirect costs among respondents with IBS-D (diagnosed and undiagnosed) and controls^a^. ^a^Includes employed respondents only (*n* = 557 for respondents with IBS-D, *n* = 33,414 for controls).*Difference significant at *p* < 0.05, adjusted for age, gender, income, education, body mass index, smoking status, alcohol use, exercise activity, and Charlson Comorbidity Index. *Abbreviations*: *IBS-D* irritable bowel syndrome with diarrhea, *USD* US dollars

IBS-D was associated with significantly increased costs resulting from both presenteeism ($5402 vs. $3518; *p* < 0.001) and absenteeism ($1642 vs. $977; *p* < 0.05). Based on overall work productivity loss, estimated indirect costs were $2486 higher per employed respondent per year for those with IBS-D compared with controls ($7008 vs. $4522; *p* < 0.001) [Fig. 3].

## Discussion

Previous research has shown the significant impact of IBS on HRQoL, work productivity, and daily activities [3, 7, 8, 13]. To our knowledge, this study is the first to demonstrate the significant burden of illness specific to the IBS-D subtype with regard to impairments in HRQoL, work and daily activities, and indirect costs, highlighting the substantial burden of IBS-D for both patients and employers.

Respondents with IBS-D in this population reported significantly lower HRQoL, including lower levels of mental and physical well-being compared with controls. Additionally, mean MCS (45.16) and PCS (47.29) scores for individuals with IBS-D were lower than the US mean norm of 50 and a standard deviation of 10 for the SF-36v2, whereas controls without IBS-D had mean scores closer to the US mean norm (MCS 49.48, PCS 50.67). The lowest scores and greatest differences from controls were seen on the general health, vitality, bodily pain, mental health, role limitations due to emotional problems, and social functioning scales, suggesting that the HRQoL burden of IBS-D is largely attributable to the mental impact of the disorder, rather than to physical limitations. These findings are in line with the previously reported impact of IBS on patients’ emotional state, including increased symptoms of anxiety and depression [14], which have been reported across IBS subtypes [15–17]. These reductions in HRQoL also underscore the negative impact IBS-D symptoms have on patients’ lives and a continued unmet need for therapeutic options to effectively treat and manage the multiple symptoms of IBS-D. The reductions in HRQoL observed among individuals with IBS-D in this study are comparable to reductions observed among patients with other chronic conditions with burdensome symptoms, such as asthma, GERD, and migraine [9, 18].

Employed respondents with IBS-D in this population reported significantly higher levels of absenteeism, presenteeism, and overall work productivity loss compared with controls, with 3.9 more missed work days per year for those with IBS-D. Respondents with IBS-D also reported greater impairments in daily activities (29.6%) compared with both controls (18.9%) and the general US population (average score of 22.1%) [19]. These impairments in work productivity and daily activities are comparable to those reported for other chronic disorders, such as among patients with controlled asthma [20].

Lost time from work translated into indirect costs of $2486 more per employed respondent with IBS-D per year in this population, with approximately three-quarters of the total annual indirect costs for respondents with IBS-D attributable to presenteeism. This suggests patients may be impacted by the chronic symptoms associated with IBS-D in a manner that impairs their ability to maintain high levels of productivity in the workforce. This productivity loss and activity impairment further emphasizes the significant impact of IBS-D symptoms on both patients and the wider society, outside of any direct medical costs that may also be incurred due to the disorder.

### Limitations

The results of this study should be interpreted in light of certain limitations. As with any survey, the data are self-reported and cannot be independently verified by respondents’ medical charts or other objective data. The data are also cross-sectional in nature and therefore do not allow for causal inferences to be made. The data extrapolated for the analysis of indirect costs represent the most up-to-date unit cost information available at the time of the study; however, this is unlikely to affect relative comparisons between groups and may even result in a more conservative estimate of the indirect costs. Finally, although a number of respondent demographic and health characteristics were controlled for, there may be additional variables that were not controlled for, which could have affected the results.

## Conclusions

This study highlights the substantial disease burden among patients living with IBS-D, a chronic functional gastrointestinal disorder characterized by bothersome bowel and abdominal symptoms. Compared with controls, respondents with IBS-D reported significantly greater reductions in HRQoL, greater impairments in work productivity and daily activities, and higher rates of absenteeism and presenteeism that translate into a significant burden to employers in terms of indirect costs. These findings suggest a significant unmet need exists for therapies to effectively treat the symptoms of IBS-D and alleviate the considerable societal and patient burden associated with this condition.

## Abbreviations

*CCI*: Charlson Comorbidity Index
*GERD*: Gastroesophageal reflux disease
*HRQoL*: Health-related quality of life
*IBD*: Inflammatory bowel disease
*IBS*: Irritable bowel syndrome
*IBS-C*: Irritable bowel syndrome with constipation
*IBS-D*: Irritable bowel syndrome with diarrhea
*MCS*: Mental Component Summary
*NHWS*: US National Health and Wellness Survey
*OTC*: Over the counter
*PCS*: Physical Component Summary
*SD*: Standard deviation
*SE*: Standard error
*SF-36*: Short Form 36 Health Survey
*SF-36v2*: Short Form 36 Health Survey version 2
*SF-6D*: Short Form-6 dimension
*USD*: US dollars
*WPAI:GH*: Work Productivity and Activity Impairment Questionnaire: General Health version.
